# Comparing the effectiveness of gratitude intervention and behavioral activation technique in breast cancer patients: a randomized trial

**DOI:** 10.1186/s12885-025-14492-3

**Published:** 2025-07-28

**Authors:** Hanie Shariatmadari, Zahra Taheri-Kharameh, Noora Kamaliuon, Bahram Ali Ghanbari Hashem Abadi, Mahmoud Shokouhi-Tabar

**Affiliations:** 1 Student Research Committee, Qom University of Medical Sciences, Qom, Iran; 2 Department of psychology, faculty of psychology and educational science, Allameh Tabataba’i University, Tehran, Iran; 3 Spiritual Health Research Center, Qom University of Medical Sciences, Qom, 3714852833 Iran; 4 Department of Public Health, School of Health, Qom University of Medical Sciences, Qom, Iran; 5 Payam Noor Aran and Bidgol University, Kashan, Isfahan, Iran; 6 Department of Psychology, School of Psychology, Ferdowsi University of Mashhad, Mashhad, Iran; 7 School of Health and Religion, Qom University of Medical Sciences, Qom, Iran

**Keywords:** Breast cancer, Gratitude, Behavioral activation, Anxiety, Depression, Mental health, Supportive care

## Abstract

**Background:**

Breast cancer diagnosis and treatment can lead to significant psychological distress, including anxiety and depression. Positive psychology interventions, such as Gratitude Intervention (GI) and Behavioral Activation (BA), have shown promise in managing mental health issues. This study aims to evaluate and compare the effects of GI and BA on anxiety and depression levels among women with breast cancer.

**Method:**

This study randomly assigned 45 breast cancer patients from a hospital in Mashhad, Iran, into two groups: the gratitude group (*n* = 22) and the BA group (*n* = 23). The participants underwent six weekly 45-minute sessions of their respective interventions. The Hospital Anxiety and Depression Scale (HADS) was employed to assess anxiety and depression levels prior to and following the intervention. Statistical analyses were conducted to ascertain the relative effectiveness of the two. methods.

**Results:**

Following the intervention, both the GI and BA groups exhibited a statistically significant reduction in anxiety scores. However, neither intervention led to a statistically significant reduction in depression scores. The mean anxiety scores in the GI group decreased from 19.54 ± 3.63 to 17.00 ± 1.79, while in the BA group, anxiety scores decreased from 21.43 ± 3.65 to 18.73 ± 2.94. The BA group demonstrated a greater reduction in anxiety scores compared to the GI group, with a mean change of 2.70 ± 0.71 versus 2.54 ± 1.84, respectively. This difference was statistically significant (*p* =.022).

**Conclusion:**

The study suggests that both gratitude exercises and BA may be beneficial in mitigating anxiety among breast cancer patients, with BA demonstrating potentially greater efficacy. However, no significant changes were observed in depression scores. Future studies should include patients with higher levels of depression and longer interventions to more accurately measure the effects of these interventions on depression.

**Trial registration:**

This trial has been retrospectively registered with the Iranian Registry of Clinical Trials (IRCT) under the registration number IRCT20241020063430N1. The registration date is November 21, 2024. The registration can be verified on the IRCT website at [https//irct.behdasht.gov.ir].

## Background

According to statistics from the World Health Organization, 2.3 million women worldwide were diagnosed with breast cancer in 2022, resulting in 685,000 deaths [1]. Projections indicate that the global burden of breast cancer will increase to over 3 million new cases and 1 million deaths by 2040 [2]. In parallel with global trends, breast cancer is also the most prevalent cancer among women in Iran, accounting for 28.1% of all female cancers in 2020 [3]. The rising incidence of breast cancer has significant psychological implications. Studies have demonstrated an association between the increasing number of cancer cases and the development of psychological disorders in patients with this disease [4]. The definitive diagnosis, periodic hospitalizations, and exhausting long-term treatment methods place cancer patients and their families under constant stress, potentially leading to psychological disorders.

It is well-documented that anxiety and depression are prevalent among cancer patients. At least one-third of individuals diagnosed with cancer present with comorbid psychiatric disorders‏ [5]. Anxiety and depression are prevalent psychological disorders observed in breast cancer patients [6]. Depression and anxiety can adversely impact physiological functioning, reduce treatment adherence, and diminish quality of life in patients with breast cancer; moreover, these psychological conditions may serve as significant determinants of survival in this population [7]. The evidence presented supports the conclusion that effective interventions are necessary to address anxiety and depression in this population.

Numerous interventions have been proposed to address anxiety and depression in individuals facing serious illness. This study focuses on two such interventions—gratitude practice and behavioral activation—comparing their efficacy in anxiety and depression among women diagnosed with breast cancer. Gratitude is defined as the human capacity to acknowledge positive aspects of life and is characterized as a positive emotional response to receiving or conferring a benefit from another individual [8]. Behavioral activation, on the other hand, is a structured evidence-based psychotherapy for depression that aims to increase engagement in meaningful activities. It operates on the principle that engaging in meaningful behaviors can impact emotions and break the cycle of depression [9].

These interventions were selected for comparison due to their distinct yet potentially complementary mechanisms for alleviating psychological distress. Gratitude practice, a cognitive-emotional intervention, aims to enhance positive affect and broaden attentional focus towards positive life aspects, which can counteract the negativity bias often seen in anxiety and depression. Conversely, behavioral activation is a behavioral approach that directly targets the anhedonia and avoidance behaviors characteristic of depression and often comorbid with anxiety by promoting engagement in rewarding and value-consistent activities. While both interventions have demonstrated potential for enhancing psychological well-being and improving mental health [10, 11], particularly within breast cancer populations [12], there is a paucity of research directly comparing their efficacy. For instance, previous research has typically assessed either gratitude interventions [13] or behavioral activation [14] as standalone treatments for psychological distress in cancer patients. However, to our knowledge, comparative studies evaluating the relative benefits of these two approaches—especially in oncology populations—remain scarce.

Understanding whether a predominantly cognitive-emotional approach (gratitude) versus a behavioral approach (behavioral activation) offers differential benefits, or if they are similarly effective for this specific population, is crucial for tailoring interventions. Furthermore, their accessibility, cost-effectiveness, and ease of implementation make them promising candidates for broader application, and a direct comparison can help care providers make more informed decisions based on individual patient needs and contextual factors.

Accordingly, this study seeks to fill this gap by exploring their impact on anxiety and depression levels in women diagnosed with breast cancer. The findings of this study will contribute to the growing body of evidence on effective psychological interventions for cancer patients and may inform future clinical practices in oncology care.

The primary objective of this study is to compare the effectiveness of gratitude intervention and behavioral activation technique in reducing anxiety and depression levels among women with breast cancer. Specifically, we aim to:

1. Evaluate the impact of gratitude intervention on anxiety and depression levels in breast cancer patients.
2. Assess the effect of behavioral activation technique on anxiety and depression levels in breast cancer patients.
3. Compare the effects of gratitude intervention versus behavioral activation technique on anxiety and depression levels among breast cancer patients.

## Method

### Study design, setting, and sample

This study employed a parallel-group randomized controlled trial design, featuring pre-test and post-test assessments. The trial was conducted between July and October 2023 at the Hope Clinic affiliated with Mashhad University of Medical Sciences, a renowned city in northeastern Iran.

A total of 45 patients diagnosed with breast cancer participated in the study. Participants were randomly allocated to either the Gratitude Intervention (GI) group (*n* = 22) or the Behavioral Activation (BA) group (*n* = 23). The random allocation sequence was generated by an independent researcher not involved in the recruitment or intervention delivery, using a computer-generated random number sequence and block randomization with a block size of 4 to maintain balance between the groups.

Participants were enrolled by a research coordinator responsible for informing them about the study and obtaining their written informed consent. The group assignment was revealed to each participant only after they had signed the informed consent form and completed the baseline assessments.

### Compliance with reporting guidelines

This study adheres to the Consolidated Standards of Reporting Trials (CONSORT) guidelines for reporting randomized controlled trials. A completed CONSORT checklist has been included as an additional file with this manuscript submission.

### Sample size

The sample size was estimated using data from a previous study [15], with anxiety as the primary outcome variable. Based on the reported mean difference of approximately 0.47 in anxiety scores (Cohen’s d ≈ 0.8, indicating a large effect size), and a standard deviation of around 0.58, the required sample size was calculated using G*Power software. With a two-tailed test, 95% confidence level (α = 0.05), and power of 90%, a minimum of 19 participants per group was required. To account for an estimated 20% attrition rate, the final target sample size was increased to 46 participants (23 per group).

### Study population

The study population comprised adult women aged 18 years or older with a medically confirmed diagnosis of stage I breast cancer. Inclusion criteria were: no prior chemotherapy and absence of diagnosed mental disorders. Participants were excluded from the study if they met any of the following criteria: withdrawal of consent, occurrence of significant adverse events (e.g., serious illness or injury impacting intervention continuation), initiation of chemotherapy during the study period (to avoid potential confounding effects), or non-adherence to the intervention protocol (defined as missing three or more of the six scheduled sessions).

### Recruitment and procedure

Patients who met the criteria for the intervention were informed about the study and enrolled by a research coordinator after giving their written informed consent. The interventions were conducted in person by a skilled psychologist who holds a master’s degree in psychology and is a mental health expert at the Omid Mashhad Hospital. The psychologist has 7 years of clinical work experience at the Omid Mashhad Cancer Hospital, with a specific interest in positive psychology interventions.

The 45-minute weekly sessions were delivered over six weeks, and participants’ completion of assigned homework was followed up via telephone. These sessions included lectures, group discussions, and Q&A segments. Participants were also provided with personalized worksheets to work on during the week between sessions.

Throughout the intervention period, intervention delivery was monitored to ensure adherence to the established protocols and the interventions were implemented as planned. All participants completed all scheduled sessions, and their adherence to assigned homework was tracked through self-report during follow-up telephone calls.

### Data collection

Before the intervention began, baseline data was collected, which included demographic information and levels of anxiety and depression assessed using The Hospital Anxiety and Depression Questionnaire. Immediately after the completion of the six-week intervention, participants were asked to complete the same anxiety and depression questionnaire again. All assessments were conducted by a research assistant.

### Interventions content

The Gratitude Intervention (GI) and Behavioral Activation (BA) technique were structured into six sessions each, conducted weekly. The GI group participated in activities aimed at cultivating a sense of gratitude, with specific objectives outlined in Table 1. Participants were given worksheets to document daily positive events, reflect on their causes, and recognize the roles of themselves and others in these occurrences. This intervention is grounded in the theoretical framework provided by Wood et al. [16].

**Table 1:** Summary of gratitude intervention contents in breast cancer patients

Session	Content
Session 1: Initial meeting	Objectives for this session included facilitating familiarity among participants, clarifying expectations, declaring confidentiality, and defining the obligations of the parties. The purpose of the sessions was stated, and participants were guided in exploring aspects of life where gratitude could be cultivated. The primary exercise involved participants receiving a pre-designed gratitude worksheet and instructions to complete it daily throughout the week. The worksheet prompted participants to list at least three to five things they were grateful for each day, briefly explain why they were grateful for each item, and identify who or what contributed to these positive aspects.
Session 2: Cultivating gratitude for blessings	This session began with a review of the previous week’s completed worksheets, allowing participants to share their experiences and insights. The discussion then focused on material blessings (e.g., wealth, health) and spiritual blessings (e.g., relationships, inner peace). Participants were encouraged to reflect on how these blessings manifested in their lives and how recognizing them fostered feelings of gratitude. Participants were instructed to continue completing the gratitude worksheet for the following week, focusing on identifying and appreciating these different categories of blessings.
Session 3: Deepening self-awareness	The third session involved reviewing the completed worksheets from the previous week and a training component focused on enhancing self-awareness related to gratitude. This training involved guided reflection and discussion on how acknowledging gratitude impacts one’s thoughts, emotions, and behaviors. Participants were encouraged to pay closer attention to their internal responses when practicing gratitude. They were instructed to complete gratitude worksheets during the week with a focus on deeper reflection on the feeling of gratitude associated with each item listed.
Session 4: Consciously seeking gratitude	Discussions in this session centered on experiences from the previous week’s worksheet and training on consciously seeking gratitude in everyday life. Participants were taught techniques for actively noticing and appreciating small, often overlooked, instances of life’s benevolence throughout their day. This involved mindful observation and reframing perspectives to identify positive aspects even in challenging situations. Participants were instructed to continue completing the gratitude worksheets throughout the week, making a deliberate effort to identify novel or subtle sources of gratitude.
Session 5: Patience and higher power	This session reviewed the progress of gratitude practice based on the previous week’s worksheet. The session included teaching about patience and seeking assistance from God (as applicable to participants’ beliefs) in their gratitude journey. Discussions explored how patience facilitates the cultivation of gratitude and how connecting with a sense of something larger than oneself—namely, God—can deepen feelings of appreciation. Participants were instructed to continue completing the gratitude worksheet for the following week, potentially incorporating reflections on patience and their connection to God.
Session 6: Continuous practice and review	The final session involved discussing and reviewing the previous week’s worksheet. The session addressed common errors related to gratitude practice and ways to avoid them. This included discussions on potential barriers to gratitude (e.g., focusing on negatives, comparing oneself to others) and strategies for overcoming them. The six sessions were summarized, emphasizing the importance of continuous gratitude practice beyond the intervention period and discussing its potential long-term effects on well-being. Participants were instructed to complete gratitude worksheets for the forthcoming week, reinforcing the habit of daily gratitude reflection.

On the other hand, the BA group focused on tracking and analyzing daily activities to promote positive experiences. Participants were tasked with developing a system for logging daily activities and using it to highlight positive experiences throughout the week. Detailed descriptions of the BA intervention are provided in Table 2. This approach is informed by prior research on behavioral activation techniques, including the study by Brief et al. [17], which demonstrates the efficacy of such methods in enhancing emotional well-being among depressed breast cancer patients.

**Table 2:** Summary of behavioral activation techniques contents in breast cancer patients

Session	Content
Session 1: Initial session	This session aimed to establish a therapeutic relationship, primarily through getting to know each other, stating the purpose of the sessions, and the client signing a simple behavioral contract. The core focus was on introducing and training the client in Behavioral Activation principles. Clients learned to track and record their daily thoughts, feelings, and behaviors using a structured worksheet. The exercise for the week involved completing the BA Worksheet daily, noting activities, associated mood levels, and any thoughts or feelings that arose.
Session 2: Understanding thoughts, emotions, and behaviors	The second session began with a review of the previous week’s completed BA worksheets, discussing patterns observed. The session included an explanation of the reciprocal relationship between thoughts, emotions, and behaviors, particularly in the context of illness. Discussions explored how inactivity or avoidance can perpetuate negative mood states and how increasing engagement in positive activities can improve emotional well-being. The impact of interpersonal interactions and drawing on others’ positive experiences were also discussed. Participants were instructed to continue filling out the BA Worksheet for the following week, paying specific attention to the link between their activities and their mood.
Session 3: Introducing positive reinforcement	This session involved reviewing the completed BA worksheet from the previous week. A key component was the introduction of verbal positive reinforcement techniques, specifically focusing on positive self-talk. Participants were guided on how to identify and challenge negative self-statements and replace them with more positive and realistic affirmations related to their activities and progress. The practical exercise for the following week involved completing the BA worksheet while actively practicing positive self-talk and noting its effect on their mood.
Session 4: Developing coping skills	In the fourth session of the Behavioral Activation (BA) intervention, participants reviewed their previous worksheet to reinforce the link between behavior and mood. The session focused on identifying and strengthening coping strategies as adaptive behaviors that align with BA’s goal of reducing avoidance and increasing engagement in meaningful activities. Participants explored coping methods such as problem-solving, seeking social support, and participating in pleasurable or value-driven actions, all of which promote psychological resilience and emotional well-being. The session also addressed how avoidance sustains distress and emphasized gradual exposure to feared or avoided situations to disrupt patterns of withdrawal. For home practice, participants were instructed to continue using the BA worksheet and deliberately implement the coping strategies, tracking their emotional responses and motivation.
Session 5: Problem-solving techniques	In this session, participants reviewed the previous week’s BA worksheet and were introduced to systematic problem-solving techniques as a core component of the Behavioral Activation model. Problem-solving is conceptualized in BA as an active, goal-directed behavior that counters the passivity and avoidance often associated with depression and anxiety. By equipping individuals with structured steps to tackle real-life challenges, this approach fosters a sense of efficacy and control, thereby enhancing engagement with life and reducing emotional distress. The session provided step-by-step instruction in the problem-solving process, which included: (1) clearly defining the problem; (2) gathering and analyzing relevant information to identify underlying causes; (3) setting realistic and measurable goals; (4) generating multiple possible solutions; (5) evaluating and selecting the most appropriate strategy; (6) developing a concrete action plan; (7) monitoring outcomes; and (8) reflecting on the process for future improvement. Participants practiced applying these techniques to their own life challenges, reinforcing BA’s emphasis on activating purposeful, adaptive behavior. For home practice, they were instructed to continue completing the BA worksheet while actively integrating the problem-solving steps into daily life, thereby reinforcing behavioral change through practical application.
Session 6: Prevention and review	The final session involved reviewing the previous week’s BA worksheet. Discussions centered on preventive measures to maintain the gains made during the intervention and avoid relapse into inactivity or negative patterns. Strategies for continuing to use behavioral activation principles in daily life were emphasized. The contents of all six sessions were summarized, reinforcing the key concepts and skills learned. The concluding exercise involved completing the BA worksheet for the forthcoming week, encouraging the continuation of activity tracking and engagement as a long-term strategy for well-being.

No significant changes were made to the study methods, eligibility criteria, or protocol after the trial commenced. The study was conducted as initially planned and registered.

### Similarity of interventions

Both the Gratitude Intervention (GI) and Behavioral Activation (BA) technique shared several structural similarities to ensure comparability. Each intervention consisted of six 45-minute weekly sessions, delivered in person by the same skilled psychologist. Both interventions incorporated lectures, group discussions, and Q&A segments. Participants in both groups were provided with personalized worksheets for homework between sessions, and their completion was followed up via telephone. The interventions were similar in their focus on daily activities and experiences, with GI emphasizing positive events and gratitude, while BA concentrated on tracking and analyzing daily activities to promote positive experiences. This structural similarity allowed for a more direct comparison of the specific content and approach of each intervention.

### Instruments

The study utilized various instruments, including demographic information and assessments of anxiety and depression levels conducted through The Hospital Anxiety and Depression Questionnaire.

1. Demographic information: This section includes details such as age, occupation, education, marital status, and insurance coverage.

2. The Hospital Anxiety and Depression Questionnaire (HADS), developed by Sigmon and Snaith in 1983, was utilized to evaluate the levels of anxiety and depression displayed by the participants, as noted by Lam et al. in 2006 [18]. This instrument consists of 14 self-report items designed to gauge the presence and severity of depression and anxiety symptoms experienced by patients over the past week. The questionnaire is divided into two subscales: one for depression and one for anxiety, each containing seven items. Participants rate each item on a scale from zero to three, with scores on the Depression and Anxiety subscales ranging from 0 to 21. Interpretation of scores is as follows: 0 to 7 indicates normal levels, 8 to 10 suggests mild symptoms, 11 to 14 points to moderate symptoms, and 15 to 21 signifies severe symptoms. The validated Persian version of the HADS was used in this study to ensure linguistic and cultural appropriateness for the Iranian population. The reliability and validity of the Persian version of this questionnaire were confirmed by Kaviani et al. in 2009 [19]. The Cronbach’s alpha coefficients for the depression and anxiety subscales were calculated to be 0.70 and 0.85, respectively.

### Statistical analysis

In this study, we conducted a thorough statistical analysis to examine the impact of the interventions on anxiety and depression scores. Descriptive statistics, including mean and standard deviation, were calculated for each variable at baseline and post-intervention. Prior to conducting parametric tests, the assumption of normality was assessed using the Shapiro-Wilk test for all outcome variables within each group. As the data met the assumption of normality, parametric tests were deemed appropriate. Independent t-tests were used to compare outcomes between the GI and BA groups. Within-group comparisons (pre- and post-intervention) were performed using paired t-tests. In addition, effect sizes (Cohen’s d) were calculated to evaluate the magnitude of the intervention effects on anxiety and depression scores.

### Ethical considerations

The study was granted ethical approval by the Qom University of Medical Sciences (IR.MUQ.REC.1402.118). Prior to participating in the study, all participants provided written informed consent. The privacy and confidentiality of participants were rigorously maintained throughout the study. Individuals who did not meet specific exclusion criteria were not included in the study.

## Results

A total of 45 breast cancer patients were included in the study, with 22 participants in the GI group and 23 participants in the BA group. The mean age of the participants was 45.83 ± 10.42 years. At baseline, there were no statistically significant differences between the Gratitude (*n* = 22) and Behavioral Activation (*n* = 23) groups in terms of age, marital status, employment, place of residence, insurance coverage, or baseline scores of anxiety, depression, and total HADS (*p* >.05 for all) (Table 3).

**Table 3:** Baseline characteristics of participants by gratitude and behavioral activation intervention groups

Variable	Gratitude (*n* = 22)	Behavioral Activation (*n* = 23)	*P*-value
Age (years), mean ± SD	44.5 ± 12.5	47.0 ± 8.2	0.439
Marital status, n (%)			0.134
Married	18 (81.8%)	19 (82.6%)	
Widowed	3 (13.6%)	3 (13.0%)	
Divorced	1 (4.5%)	0 (0.0%)	
Employment status, n (%)			0.903
Unemployed	11 (50.0%)	12 (52.2%)	
Employed	5 (22.7%)	6 (26.1%)	
Place of residence, n (%)			0.570
City	16 (72.7%)	20 (87.0%)	
Village	3 (13.6%)	3 (13.0%)	
Insurance status, n (%)			0.329
Yes	17 (77.3%)	20 (87.0%)	
No	2 (9.1%)	2 (8.7%)	
Anxiety, mean ± SD	10.8 ± 3.1	12.1 ± 2.7	0.433
Depression, mean ± SD	8.6 ± 1.5	9.3 ± 1.1	0.517
HADS total, mean ± SD	19.5 ± 1.9	21.4 ± 2.7	0.380

Percentages may not add up to 100% due to rounding

Within-group analysis revealed that both the GI and BA groups experienced statistically significant reductions in anxiety scores following the intervention (GI: *p* =.001, d = 0.84; BA: *p* =.003, d = 1.04), with large effect sizes observed in both groups. In contrast, changes in depression scores were not statistically significant in either group (GI: *p* =.373, d = 0.28; BA: *p* =.729, d = 0.07), indicating small to negligible effects.

Regarding the overall HADS score, both groups demonstrated statistically significant improvements post-intervention. The mean score decreased from 19.54 ± 3.63 to 17.00 ± 1.79 in the GI group (*p* =.009, d = 0.89) and from 21.43 ± 3.65 to 18.73 ± 2.94 in the BA group (*p* =.007, d = 0.81), both indicating large effect sizes.

Between-group comparisons of post-intervention outcomes are presented in Table 4. Although no statistically significant differences were observed between the groups for anxiety (*p* =.135) or depression (*p* =.133), the reduction in total HADS score was significantly greater in the BA group compared to the GI group (*p* =.022).

**Table 4:** Comparison of outcome measures before and after intervention within groups

Outcome	Group	Before Intervention Mean ± SD	After Intervention Mean ± SD	Mean Difference (95% CI)	*P*-value	Cohen’s d
Anxiety	Gratitude	10.86 ± 3.16	8.72 ± 1.69	2.13 (0.98, 3.21)	0.001	0.84
	Behavioral Activation	12.08 ± 2.77	9.56 ± 1.99	2.52 (0.96, 3.99)	0.003	1.04
Depression	Gratitude	8.68 ± 1.96	8.20 ± 1.50	0.40 (–0.52, 1.34)	0.373	0.28
	Behavioral Activation	9.34 ± 2.74	9.17 ± 2.18	0.17 (–1.06, 1.50)	0.729	0.07
HADS	Gratitude	19.54 ± 3.63	17.00 ± 1.79	2.54 (0.70, 4.38)	0.009	0.89
	Behavioral Activation	21.43 ± 3.65	18.73 ± 2.94	2.69 (0.83, 4.55)	0.007	0.81

No adverse events or participant dropouts were reported in either group throughout the study period.

### Participant flow and losses

All 45 participants completed the study and were included in the analysis. There were no losses or exclusions after randomization in either group.

## Discussions

This study aimed to investigate the effects of gratitude and behavioral activation (BA) on anxiety and depression among women with breast cancer. The results suggest that while both the gratitude and BA groups experienced a significant reduction in anxiety scores, the reduction in depression scores did not reach statistical significance in either group.

The significant reduction in anxiety scores in both groups aligns with previous research. For instance, Tomczyk et al. (2021), in a study on women with breast cancer, found that gratitude is positively associated with well-being and effective coping strategies, leading to reductions in both depression and anxiety [13]. Similarly, Sztachańska et al. (2019) also focusing on women with breast cancer, demonstrated that gratitude can enhance daily psychological functioning and support adaptive coping, further corroborating our findings [12]. Rooyni and Voskooly (2013) investigated the correlation between gratitude, well-being, and distress in patients with breast cancer, revealing a negative correlation between gratitude and anxiety, hostility, and depression [20]. A recent meta-analysis similarly indicates that gratitude interventions can help prevent or reduce depression, emphasizing the potential benefits of gratitude in reducing anxiety and depression in women with cancer [21].

In terms of BA, Consistent with our results, the study by Hopko et al. (2016) demonstrated that behavioral activation can reduce anxiety in patients with breast cancer [14]. Furthermore, a randomized clinical trial conducted by Hopko et al. (2011) indicated that behavioral activation was effective in decreasing anxiety levels among women with breast cancer [17]. Similarly, a case study by Armento et al. (2009) reported the positive impact of a behavioral activation intervention [22]. Berg et al. (2023), which suggests that BA is effective for reducing anxiety in adults with generalized anxiety disorder [23].

However, our study’s finding that BA did not significantly impact depression contrasts with other research in this area. For example, a recent narrative review demonstrated the efficacy of BA in treating major depressive disorder [24]. In another narrative review of the empirical literature in 2022, Alber et al. also confirmed the effectiveness of BA as an intervention for major depressive disorder [10]. Malik et al. (2021) also provided evidence supporting BA’s effectiveness for depression [25]. Several explanations may be proposed for this unexpected finding. The initial depression levels of participants (pre-intervention) in both groups, as measured by the HADS questionnaire, were mild: Gratitude (8.68 ± 1.96) and Behavioral Activation (9.34 ± 2.74). This baseline condition may have limited the potential for improvement, possibly leading to statistically insignificant results. Furthermore, the six-week intervention period may not have been sufficiently long for individuals with mild depression to manifest significant changes in depressive symptoms. Additionally, this study specifically focused on breast cancer patients, who may possess unique psychological needs and responses compared to other populations studied in the literature.

Breast cancer patients often face a complex interplay of physical illness, existential concerns, body image issues, and treatment-related fatigue, which may reduce the effectiveness of generic depression interventions such as BA. In this context, symptom relief may depend more on addressing cancer-specific emotional challenges than solely increasing engagement in behavioral activities. Moreover, the psychological adaptation process in cancer patients often involves grief, fear of recurrence, and role changes, which may not be adequately targeted by standard BA protocols. Therefore, the lack of significant effect may suggest a need to tailor BA more specifically to the cancer context—perhaps by integrating psycho-oncological components or focusing on illness-specific goals and values.

Healthcare providers are encouraged to integrate gratitude practices into standard care for breast cancer patients. Simple activities such as writing thank-you notes or other forms of gratitude expression can significantly improve mental health. Additionally, BA techniques may be beneficial as part of psycho-oncology interventions, helping patients engage in meaningful activities, improve mood, and reduce anxiety symptoms. Considerations regarding the availability and cost-effectiveness of these services are crucial for breast cancer patients.

The potential mechanisms through which gratitude may reduce anxiety and depression include redirecting the brain’s attention from negative to positive thoughts, acting as a protective factor against mental health challenges, and improving interpersonal relationships and overall well-being [26, 27]. For BA, the mechanisms include breaking the cycle of negative mood and low motivation, increasing exposure to natural reinforcers, enhancing self-efficacy through goal setting and problem-solving, and challenging negative thoughts and beliefs [28–30].

### Generalizability and cultural considerations

The findings of this study should be interpreted with caution regarding their generalizability. The sample consisted solely of women with breast cancer from a specific region in Iran, which may limit the applicability of the results to other populations or cultural contexts. Cultural factors, including beliefs about illness, coping mechanisms, social support structures, and attitudes toward psychological interventions, can significantly influence both the experience of psychological distress and response to treatment [31]. Thus, the efficacy of gratitude interventions and behavioral activation might vary across different cultural settings. Future research should consider diverse populations and incorporate culturally adapted interventions to enhance the external validity of these findings.

### Limitation

The present study had several limitations that should be acknowledged. The small sample size of only 45 women with breast cancer may limit the generalizability of the findings. A larger sample would improve the reliability and statistical power of the results. The interventions were carried out over a 6-week period, but longer follow-up periods could offer more insight into the lasting effects of the intervention. The study relied on self-reported measures for psychological distress, which may not be as accurate as objective measures or clinician assessments. Furthermore, the study focused exclusively on GI and BA techniques, but incorporating other complementary interventions could potentially lead to greater benefits. While the study did compare pre-test and intervention conditions, the absence of a control group hinders the ability to make causal conclusions. Including a control group in future studies would allow for a more robust comparison. Additionally, the study included participants with mild to moderate anxiety and mild depression at baseline. Future research would benefit from recruiting patients with a higher cutoff point, such as moderate to severe anxiety and depression, to further assess the interventions’ efficacy for a broader range of symptom severity. Blinding was not performed in this study, which is acknowledged as a limitation. Future studies are encouraged to implement blinding procedures wherever feasible to reduce potential sources of bias.

## Conclusion

This study provides preliminary evidence suggesting that both Gratitude Intervention (GI) and Behavioral Activation (BA) may be beneficial in reducing anxiety among breast cancer patients, with BA demonstrating potentially greater efficacy. However, the findings require further investigation with larger sample sizes and extended follow-up periods to confirm their efficacy and long-term impact. Future research should also explore the potential role of these interventions in addressing depression in breast cancer patients and investigate the potential combined effects of GI and BA. Overall, this study contributes to the growing body of literature exploring the potential of psychological interventions in managing the emotional distress associated with breast cancer, highlighting the need for further research to refine our understanding of these interventions’ effectiveness and inform the development of evidence-based supportive care programs.

## Data Availability

The data that support the findings of this study are available from the corresponding author upon reasonable request.
